# Peanut oral immunotherapy in adolescents: study protocol for a randomized controlled trial

**DOI:** 10.1186/s13063-015-0717-y

**Published:** 2015-04-29

**Authors:** Elodie Michaud, Bertrand Evrard, Bruno Pereira, Emmanuelle Rochette, Lise Bernard, Paul-Olivier Rouzaire, Nelly Gourdon-Dubois, Etienne Merlin, Jean-Luc Fauquert

**Affiliations:** CHU Clermont-Ferrand, Pole pédiatrique, Unité d’allergologie de l’enfant, CHU Estaing, 1 Place Lucie et Raymond Aubrac, F-63003 Clermont-Ferrand, France; CHU Clermont-Ferrand, Département d’immunobiologie, CHU Estaing, 1 Place Lucie et Raymond Aubrac, F-63003 Clermont-Ferrand, France; INSERM, UMR1019, F-63003 Clermont-Ferrand, France; CHU Clermont-Ferrand, Unité de Biostatistiques, Direction de la Recherche Clinique (DRCI), 58 rue Montalembert, F-63003 Clermont-Ferrand, France; INSERM, CIC 1405, CHU, F-63003 Clermont-Ferrand, France; CHU Clermont-Ferrand, Département de Pharmacie, 58 rue Montalembert, F-63003 Clermont-Ferrand, France; CHU Clermont-Ferrand, Pole pédiatrique, Service de pédiatrie générale et Multidisciplinaire, CHU Estaing, 1 Place Lucie et Raymond Aubrac, F-63003 Clermont-Ferrand, France

**Keywords:** Peanut allergy, Oral immunotherapy, Adolescent, Double blind placebo controlled oral food challenge

## Abstract

**Background:**

Peanut allergy is an increasingly common health problem. Current treatment guidelines are based on strict avoidance. However, in the last few years, oral immunotherapy protocols have shown promising results yielding increased tolerance to peanut in allergic children. Adolescence is particularly at risk.

**Methods/Design:**

We have designed a randomized, double-blind, placebo-controlled, multicenter study to investigate the efficacy and safety of peanut oral escalating immunotherapy in a 12- to 18–year-old population with proved allergy to peanut. Patients are selected when the threshold of peanut intake is over 100 mg and 2 cumulated g on the first double-blind, placebo-controlled oral food challenge (DBPCOFC).

During the build-up placebo-controlled blinded phase, doses containing peanut or placebo will be administered by gradual up-dosing from 10 mg to 2 g with 2-weekly increments. After this first randomized phase, the desensitized participants will continue to intake native peanut in an unblinded process during 13 or 37 weeks following a second randomization. Adverse events are picked up and managed throughout the entire protocol.

The main endpoint is the percentage of patients with negative DBPCOFC at the threshold of 2 g of cumulative peanut at the end of the build-up phase of 24 weeks.

Secondary endpoints include: (1) desensitization 6 weeks and 6 months after the end of the maintenance phase; (2) adverse effects during the build-up phase; (3) immunological profile confirming peanut desensitization. Immunologic assays will be carried out at every DBPCOFC and at the middle of the build-up phase to evaluate the peanut immunologic profile modifications.

**Discussion:**

This double-blind, placebo-controlled study will be, to our knowledge, the first evaluation of a peanut oral immunotherapy protocol in teenagers in the purpose to reduce severe reactions after unexpected intake and to improve quality of life.

**Trial registration:**

ClinicalTrial.gov: NCT02046083 (23 January 2014).

## Background

Peanut allergy is one of the most common forms of food allergy encountered in clinical pediatric practice especially in children above 3 years old. It affects about 1% to 2% of children in France [1]. The allergy carries a risk of acute life-threatening reactions even after the ingestion of small amounts of peanut [2]. Peanut allergy spontaneously resolves in about 20% of children only [3] and thus current guidelines for allergy management consist of strict avoidance of the allergen. Such a diet is challenging for children and their families because of the widespread presence of peanut in food preparations. Hence, in a study of Swedish children, despite theoretical strict avoidance, peanut was reported to be responsible for 19% of anaphylaxis reactions [4]. Anaphylaxis can be life-threatening and this risk justifies the holding of rescue medications such as self-injectable epinephrine at home and at school. Adolescents are particularly at risk because they have a high incidence of severe reactions following unwitting ingestion of peanut [5]. These reactions account for most of the fatalities caused by food-induced anaphylaxis [6]. However, adolescent behavior is unpredictable and can include consumption of alcohol, which makes it difficult to adhere to a strict avoidance diet. Several studies have indicated that early consumption of allergenic food like peanuts may be a more beneficial strategy. For example, in Israel, where infants are introduced early to peanuts, the prevalence of peanut allergy is lower in children than in the UK [7]. In adolescent populations, specific oral immunotherapy is an interesting way of modifying the natural history of peanut allergy. Since 2009, peanut-specific oral immunotherapy protocols have been implemented with the aim of inducing desensitization, which was achieved in 61% [8] to 84% of cases [9]. In a Cochrane review published in 2012 by Sheikh and Nurmatov [10], only one double-blind, placebo-controlled study [9] validated the concept of oral immunotherapy for peanut allergy in children. However, this trial involved only children aged less than 10 years, and nothing is known about desensitization in adolescents with high-level peanut specific IgE. Recently, Anagnostou *et al*. demonstrated the interest of tolerance induction versus strict avoidance [11].

Our aim was to assess the efficacy and safety of oral immunotherapy in a teenage population at risk of severe reactions to peanut in order to improve their quality of life and enable them to eat freely in any restaurant and in the school canteen. We also decided to investigate the length of the maintenance phase needed to induce long-term desensitization.

## Methods/Design

### Objectives and design

The main objective is to investigate in an adolescent population the efficacy and safety of peanut oral escalating immunotherapy up to a maximum level of 2 g of peanut. The secondary objectives are to compare two maintenance durations (13 and 37 weeks) with 2 g daily of peanut intake, to investigate the immunologic effects of peanut oral immunotherapy, and to assess the safety of the procedure.

**Figure 1:**
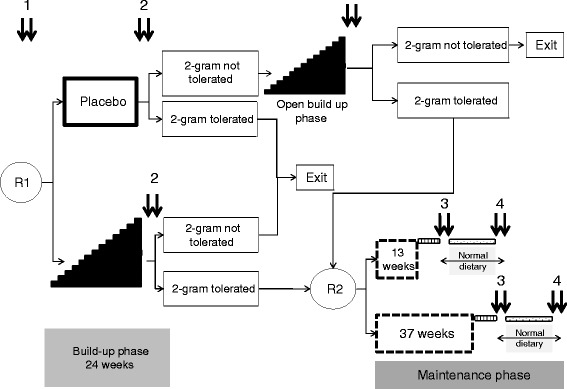
Study design. Children aged 12 to 18 years with peanut allergy proven in a first double-blind placebo-controlled oral food challenge (DBPCOFC) will enter a double-blind placebo-controlled induction phase through a first 2:1 randomization (R1). After a successful build-up phase, the maintenance phase duration will be determined by a second 1:1 randomization (R2). Double arrow: DBPCOFC. (dotted area: 24 weeks normal dietary; shaded area : 6 weeks wash-out).

The study was designed in two phases: build-up and maintenance immunotherapy (Figure 1). After a baseline double-blind placebo-controlled oral food challenge (DBPCOFC), participants will be randomized (R1) in the build-up phase (24 weeks) to one of the two groups (treatment or placebo). At the end of the build-up phase, desensitized participants will be randomized (R2) for the maintenance phase (13 versus 37 weeks’ duration). During this second phase there will be no blinding.

A subsequent DBPCOFC will be performed 6 weeks and 6 months after the completion of the maintenance phase to evaluate the peanut’s tolerance after stopping the daily peanut’s consumption. The participants will have a normal diet (trace of peanut and small quantities of peanut) after the penultimate DBPCOFC.

### Consent statement

This peanut oral immunotherapy study is a randomized, double-blind, placebo-controlled, multicenter trial. It was approved by the Institutional Review Board of the University Hospital of Clermont-Ferrand (France). By 1 March 2013, the study had been approved for all centers by a central ethics committee (Comité de Protection des Personnes Sud-Est VI, Clermont-Ferrand, France) with the registration number RBHP-2013-Fauquert and registered at http://www.clinicaltrials.gov (NCT02046083). Before inclusion the patients (and their parents) are fully informed of the aims, procedures, safety measures, and practical aspects of the protocol. A written consent is mandatory before inclusion to confirm their agreement.

### Study population

#### Inclusion criteria

Adolescents aged 12 to 18 years with a clinical history of reaction to peanut within 60 min of ingestion or a positive DBPCOFC to peanut below 2 cumulated g will be included.

Sensitization is proved by a positive skin prick test (SPT) (3 mm over the negative control) and/or a serum specific IgE level over 12 IU/mL for peanut or over 5.8 IU/mL for Ara h2 recombinant fraction to peanut.

#### Exclusion criteria

Patients with a history of severe peanut anaphylaxis requiring hospitalization in an intensive care unit, participants with severe persistent asthma or poorly controlled atopic dermatitis, or those with another concomitant severe food allergy (egg, milk, nuts, and so on) will be excluded, as will patients affected by lactose intolerance or sunflower allergy.

Participants who tolerate more than 2 cumulated g of peanut during the initial DBPCFC or those with an allergic reaction occurring below an intake of 100 cumulated mg peanut will not be included. Patients unable to follow a daily intake of capsules or to fill in a daily recording of clinical signs were not included as well as those living more far from a reference center to assume management of an acute reaction within 15 min, and those attending another clinical trial. The occurrence of a severe adverse event will lead to exclusion by the investigator or the expert committee.

### Randomizations

After the initial DBPCOFC, attribution of active treatment (peanut) or placebo will be done by a first randomization (R1) at a ratio treatment/placebo of 2:1. Stratification will be performed every six participants. For the second randomization (R2), the ratio between the two groups will be set at 1:1. Randomizations will be stratified by location due to the multisite nature of the study.

Each patient will be identified by an identification number consisting of the number of permanent investigation center and an individual patient number. The patient number will be assigned chronologically on the randomization list. A comprehensive document describing the randomization procedure will be kept as confidential at the Children Clinical Research Centre, INSERM CIC 1405.

### Procedures and interventions

Adolescents will be recruited from the pediatric allergy unit in the University Hospital, Clermont-Ferrand, France and from the pediatric departments of Saint-Etienne and Lyon hospitals.

In an education session, the patients and their parents will learn how to recognize food allergy symptoms, to evaluate their severity, and to use the rescue medications and self-injectable epinephrine.

A first DBPCOFC is managed according to national recommendations [12]. It consists in the intake of incremental doses of peanut or placebo according to a randomized plan. A food challenge is declared positive as soon as an objective reaction occurs. At this time discontinuation and management of the clinical reaction should be considered [13]. Blind is released after the second day of challenge. Patients with a peanut allergy threshold above 100 mg and below 2 g cumulative intake of peanut are included in the build-up phase.

During the build-up phase, doses will be administered by gradual up-dosing from 10 mg to 2 g of peanut with 2-weekly increments. Experimental treatment will consist in administration of doses of either peanut or placebo provided by the pharmacy department. The raw material of the peanut treatment is a mixture of peanuts (95.7%) and sunflower oil (4.3%), supplied by MENGUYS ©. A certificate of alimentary confirmed the food as suitable for human diet. The doses contain 10 mg to 500 mg of peanut. Placebo will be similarly prepared and comprise the same raw material except for the peanut paste. The participants should take the treatment in the morning. Practice of any sport is prohibited for 2 h. During the protocol, adverse events and rescue medications are picked up in the patient’s paper diary. They are quoted according to Ring and Messmer classification [13]. In the event of a grade 1 reaction occurring during the build-up phase, the increments process will not be modified. If a grade 2 or 3 without anaphylaxis reaction occurs, the level of peanut intake may be maintained for three additional periods of 2 weeks, that is, for a maximum duration of 8 weeks. In all cases symptomatic drugs will be prescribed, depending on the severity of the reaction, H1 antihistamines in cases of a mild reaction (urticaria, rhinitis, conjunctivitis, oral syndrome, acute abdominal pain, and so on), inhaled betamimetics in cases of bronchospasm, and systemic steroids and epinephrine in cases of grade 3 or 4 reaction (systemic anaphylaxis, anaphylactic shock). In the event of a grade 3 adverse reaction without anaphylactic shock, disruption should be considered. Grade 3 with anaphylactic shock and grade 4 reactions should lead to questioning the independent committee and excluding the patient. During the entire protocol, the patients are allowed to take any medication including inhaled steroids and antihistamines if needed.

During the maintenance phase, children will receive a 2 g package of crushed peanut of the same origin. Clinical follow-up is performed in the investigation center every 4 weeks. A third DBPCOFC is performed 6 weeks after the end of this maintenance phase of 13 versus 37 weeks’ duration, depending on second randomization. Finally, a fourth DBPCOFC is performed after a 6-month period where diet is free, peanut intake is freely recommended. During the entire protocol, participants will follow a diet consisting of avoidance of peanut. A weekly meeting involving all investigators allows defining the management of adverse events and the protocol’s achievements. Discontinuation of the protocol can be decided at any time by the investigator or the patient, and is mandatory when ordered by the independent data monitoring safety committee.

### Complementary investigations

#### In vivo tests

Skin prick tests to peanut will be performed before each DBPCOFC, including a positive (histamine hydrochlorhydre 10 mg/mL) and a negative (saline solution) control.

#### In vitro tests

The immunological profile will be monitored during the protocol before each DBPCOFC. Assays include total IgE and IgG 4 levels, peanut specific IgE and IgG4 levels for peanut and the following recombinant fractions: rAra h1, rAra h 2, rAra h3, rAra h8, rAra h9 (ImmunoCAP®, Phadia 250®, Thermofischer®). In addition, peanut specific basophil activation and degranulation will be assessed by a basophil activation test (CCR3, CD63, and CD203c, Flow CAST® and CD203c Reagent set, Bühlmann).

In addition, specific peanut IgE and IgG4 assays will be measured midway through the build-up phase. Cross-reactions will be evaluated during the initial assessment. Assays include specific IgE levels for lupine, walnut, almond, hazelnut, pistachio, sunflower, and Cross Carbohydrate Determinants (CCD). All samples will be stored at -80°C in a serum bank approved by the Agence Nationale de Securité du Médicament (ANSM) (2012-A01040-43).

### Judgment criterion

During each DBPCFC, peanut intake will be incremented every 30 min as follows: 10 mg, 20 mg, 40 mg, 80 mg, 160 mg, 300 mg, 500 mg, 890 mg, 2 g, 5 g. DBPCOFC will be considered positive at the threshold of 2 g if a patient exhibits at least one clinical objective sign of allergic reaction (dermatological, respiratory, gastrointestinal, or systemic involvement) within 30 min following the intake. If the patient reports subjective signs, 30 min can be added. Thereafter, the cumulative intake dose will be considered as the positive threshold of the OFC.

### Study endpoints

The main endpoint is the percentage of patients who satisfy to the judgement criterion, that is, negative DBPCOFC at the threshold of 2 g of cumulative intake of peanut at the end of the build-up phase of 24 weeks.

Secondary endpoints include:

- the percentage of patients with a negative DBPCOFC at the threshold of 2 cumulated g performed 6 weeks and 6 months after the end of the maintenance phase;
- the percentage of patients who quadruplet their reaction threshold between the first and the second DBPCOFC;
- the percentage of adolescents who have adverse effects during the build-up phase (degree and number of adverse events, use of rescue medication, rescue consultations; and hospitalizations);
- immunological profile confirming peanut desensitization (skin prick tests, peanut specific IgE and IgG 4, basophil activation test).

### Blinding

Indistinguishable peanut and placebo doses will be sent by the hospital pharmacy department. Investigators will not be aware of the treatment allocation of the patient, including during the DBPCOFC. A computer program will generate the coding list and will allocate coding numbers to patients from the specific trial site. In each participating center, the data will be collected and recorded onto case report forms (CRFs) by local research coordinators blinded to the randomized intervention. A trained research coordinator, also blinded to the randomized intervention, will centralize data from all sites and will record them onto an electronic database.

### Suspension of protocol

The protocol may be suspended for an individual patient if a severe adverse event occurs at any time during the protocol or if the patient cannot complete the build-up phase because of severe or repeated allergic reactions leading to prolonged stagnation at a given step.

The data monitoring safety committee will recommend interruption of the trial if a patient’s safety is compromised.

### Statistics

For this study, 60 randomized patients are necessary to detect a 50% difference in the primary outcome, at a two-sided α level of 0.05 and a statistical power of 90%, assuming a 60% rate of desensitization after the build-up phase in the treated group and 10% in the placebo group [8,9,14]. Assuming a successful completion of desensitization of 65%, 40 patients should be entered at the second randomization. n = 20 patients by group will provide a minimal statistical power of 80% to detect a relative difference of 30% after the maintenance phase.

Statistical analysis will be conducted on an intention-to-treat basis. Fisher’s exact test will be used for primary outcome analysis. Continuous variables will be compared with the unpaired t test or the Mann-Whitney test when appropriate. Owing to sample size, non-parametric tests will often be preferred. Longitudinal analysis using random-effects models will be performed to take into account between and within subject variability. All analyses will be conducted with Stata statistical software, version 13 (StataCorp LP, College Station, TX, US). A two-sided *P* value of less than 0.05 will be considered to indicate statistical significance.

### Registration

Data will be collected and recorded onto CRFs in each center by trained local research coordinators blinded to the randomized intervention. A trained research coordinator will centralize data from all sites and will record them onto the electronic database. The data will comprise pre-randomization and baseline characteristics (gender, age, height, weight, the first reaction with peanut, any food allergy, treatments) and items recorded the day of the first DBPCFC: wheal of skin prick test, total IgE and IgG4 assays, specific IgE and IgG4 to native peanut and to the main recombinant fractions of peanut (Ara h1, Ara h2, Ara h3, Ara h8, Ara h9).

### Data handling and record keeping

Data will be handled according to French law. All original records (including consent forms, CRFs and relevant correspondence) will be archived at trial sites for 15 years. The clean database file will be anonymized and maintained for 15 years.

### Study organization and funding

The study is an investigator-initiated trial. The study will be promoted by the University Hospital, Clermont-Ferrand, France. There is no industry support or involvement in the trial.

### Duration and timeline

Patients from three French University Hospitals will constitute the study population. Trial tools (CRF, randomization system) were developed in 2013. The protocol was submitted for first approval by the ethics committee in 2013 and definitively accepted in March 2014. The 28-month inclusion period started in September 2013. The database will be ‘edited’ and finalized in 2016. The data will then be analyzed and incorporated into an article that will be submitted for publication.

## Discussion

Peanut allergy is a frequent and severe allergy in children [15]. Eighty percent of patients remain allergic at the age of 16 years. Until the last decade, the management of peanut allergy was limited to allergen avoidance but since then various interventional protocols of induced tolerance have been developed and tested. The aim of this new mode of treatment is to reduce severe reactions after unwitting ingestion of peanut and to improve quality of life [8,9,14-16]. The mechanism by which induced tolerance to food allergy works is still a subject of debate, as is the route of application of the allergen. Greater safety has been achieved since the adoption of slow increases in the increments of doses but as the treatment is still in its infancy stages it is not yet used in daily practice. In 2012 a Cochrane data analysis [10] validated only one double-blind versus placebo study. In this study, most patients were children. However, adolescents are a population at risk in terms of serious adverse events and reduced quality of life. The incidence of accidents subsequent to unexpected intake remains high [5] at this age. In addition, spontaneous resolution of peanut allergy is unlikely after the age of 12 years.

As far as avoidance remains the sole treatment, its practice is hazardous. The term ‘traces’ is commonly used on the labels of various foods. Its definition, however, remains unclear and varies according to countries. Since there are very few trials about oral immunotherapy for peanut allergy in children, there is no well-admitted method on doing such trial. In particular, the threshold to assess peanut tolerance is of importance. We chose the threshold of 2 g of peanut because it allows a security gap in relation to trace levels and is consistent with normal daily eating habits. This means that any amount over 2 g (4 peanuts), which is a common level of intake in West European countries, is not allowed. The patients who adhere to the protocol will improve their daily quality of life and reduce the risk of severe reactions after unwitting intake [17]. Inclusion criteria were drawn up on the basis of previous studies (unpublished observations): the wheal size of SPT is commonly accepted to be positive when greater than 3 mm. The threshold (PPV 100%) of specific IgE (over 12 IU/mL) and the ara h2 recombinant fraction of peanut were studied in a local population of children allergic to peanut.

Adverse events will be recorded and managed. If serious unexpected events occur, the independent security committee will decide on whether to suspend the protocol. The mechanisms involved in desensitization and tolerance induction will be investigated.

During the build-up and maintenance phases, patients will be asked to retain a strict avoidance of peanut. However, certain co-morbidity factors could interfere with the evolution of peanut allergy under peanut induction of tolerance, in particular associated food allergies, quality of control of asthma, and infectious concomitant diseases. There will be a 6-month follow-up period after the end of the protocol during which the patients will remain under observation in the outpatient clinic. We shall compare the two duration periods of the maintenance phase in our protocol with those of previous publications. The behavior of patients who are scheduled to return to a non-restricted diet is also an unknown factor. All patients will be invited to go on eating daily reasonable amounts of peanut. One cannot expect that all patients will behave similarly in terms of daily diet. DBPCOFC 4 will feed this debate.

## Trial status

Recruitment started from September 2013 to December 2015.

## Abbreviations

ANSM: Agence Nationale de Securité du Medicament
CRF: case report forms
DBPCOFC: double-blind placebo-controlled oral food challenge
OFC: oral food challenge
SPT: skin prick test
